# Cosmogenic radionuclides reveal an extreme solar particle storm near a solar minimum 9125 years BP

**DOI:** 10.1038/s41467-021-27891-4

**Published:** 2022-01-11

**Authors:** Chiara I. Paleari, Florian Mekhaldi, Florian Adolphi, Marcus Christl, Christof Vockenhuber, Philip Gautschi, Jürg Beer, Nicolas Brehm, Tobias Erhardt, Hans-Arno Synal, Lukas Wacker, Frank Wilhelms, Raimund Muscheler

**Affiliations:** 1 Department of Geology – Quaternary Sciences, Lund University, 22362 Lund, Sweden; 2 British Antarctic Survey, Ice Dynamics and Paleoclimate, Cambridge, CB3 0ET UK; 3 Alfred-Wegener-Institut Helmholtz-Zentrum für Polar- und Meeresforschung, 27570 Bremerhaven, Germany; 4 Laboratory of Ion Beam Physics, ETH Zürich, 8093 Zürich, Switzerland; 5 Department of Surface Waters, Swiss Federal Institute of Aquatic Science and Technology, 8600 Dübendorf, Switzerland; 6 Climate and Environmental Physics, Physics Institute and Oeschger Centre for Climate Change Research, University of Bern, 3012 Bern, Switzerland; 7 Department of Crystallography, Geoscience Centre, University of Göttingen, Göttingen, Germany

**Keywords:** Climate sciences, Solar physics

## Abstract

During solar storms, the Sun expels large amounts of energetic particles (SEP) that can react with the Earth’s atmospheric constituents and produce cosmogenic radionuclides such as ^14^C, ^10^Be and ^36^Cl. Here we present ^10^Be and ^36^Cl data measured in ice cores from Greenland and Antarctica. The data consistently show one of the largest ^10^Be and ^36^Cl production peaks detected so far, most likely produced by an extreme SEP event that hit Earth 9125 years BP (before present, i.e., before 1950 CE), i.e., 7176 BCE. Using the ^36^Cl/^10^Be ratio, we demonstrate that this event was characterized by a very hard energy spectrum and was possibly up to two orders of magnitude larger than any SEP event during the instrumental period. Furthermore, we provide ^10^Be-based evidence that, contrary to expectations, the SEP event occurred near a solar minimum.

## Abstract (web summary)

Cosmogenic radionuclides from ice cores and tree rings indicate that an extreme solar proton event has hit Earth about 9200 years ago. Contrary to expectations, the event occurred during a quiet phase of the Sun within the 11 year solar cycle.

## Introduction

Solar energetic particle (SEP) events occur when abrupt eruptive events on the surface of the Sun, such as coronal mass ejections (CMEs) and solar flares, accelerate particles into the interplanetary medium. These particles – mostly protons – can eventually reach the Earth guided by the heliospheric magnetic field lines.

In the last decades, great attention has been dedicated to solar storms due to the high vulnerability of our modern society to such events. SEP events can, in fact, have serious repercussions on communication and power systems, satellite life expectancy and aircraft operations. For instance, during the so-called ‘’Halloween storms” of 2003, parts of Europe were left without electricity for several hours, and transformers in South Africa were permanently damaged, with enormous costs for society^1^. In addition, the life of astronauts in space could be endangered due to high radiation exposure connected to SEPs. For example, if an Apollo mission had flown during the SEP event of August 1972, the amount of radiation experienced by the astronauts would have led to severe, possibly even lethal, consequences^2^. The Apollo missions flew in April and December of the same year.

Furthermore, SEPs have been shown to have an impact on the atmosphere and, for example, trigger ozone depletion^3–5^, with possible effects on climate^6,7^.

Before the advent of spaceborne measurements to monitor the fluxes of protons in the 1960s, instrumental observation of SEP events has been carried out since the 1950s with neutron monitors. To go further back in time, it is possible to rely on proxy data, such as cosmogenic radionuclides from ice cores and tree rings. Cosmogenic radionuclides, such as ^14^C, ^36^Cl, and ^10^Be, are produced within the Earth’s atmosphere – mainly in the stratosphere - as a result of the interactions of galactic cosmic rays (GCR) with its constituents and are modulated by the solar and the Earth’s magnetic fields. The enhanced flux of relatively lower energy particles during a SEP event can trigger additional production of cosmogenic radionuclides, leaving an imprint in environmental archives.

The strength of SEP events is commonly quantified by the fluence of particles above 30 MeV (F_30_), that is the integrated flux of particles with kinetic energy above 30 MeV per unit area. This, together with the spectral hardness – which is the proportion of higher-energy protons (>200 MeV) compared to lower-energy protons (>30 MeV) – provides a measure to characterize the events. Some of these events possess sufficient fluxes of high-energy protons (>0.5 GeV) to reach ground-based instruments such as neutron monitors, and are called ground level-enhancements (GLE). To date, GLE no.5 of February 1956 is considered to be the largest hard event detected by ground-based methods, with a F_30_ of 1.42 × 10^9^ protons/cm^2^ ^8,9^, and it is estimated to have caused an increase of about 5% in the global ^10^Be production rate^10,11^ compared to the annual GCR-induced production. However, GLE no.5 has not left any significant imprint in ^10^Be measured in firn cores^12–14^, not even in seasonal data from Greenland^15^ likely due to the inherent weather/deposition noise in the data and measurement uncertainties.

So far, three events have been detected unambiguously in ^10^Be, ^36^Cl, and ^14^C records – in 774/5 CE^16–18^, 993/4 CE^16,19,20^ and 660 BCE^21–23^. The 774/5 CE event is the largest SEP event described so far, with a F_30_ estimated to be one order of magnitude larger than the largest GLE on record (GLE no.5 – 1956-02-23). The results presented by Mekhaldi et al.^16^ and O’Hare et al.^21^, indicate that the discovered events were significantly larger than the SEP events detected since the 1950s, thus implying a so far underestimated threat to our society.

Here we present high-resolution ^10^Be and ^36^Cl data from the NGRIP (Northern Greenland Ice core Project, 75°6′N, 42°19′W, 2917 m a.s.l.) ice core at 1-year and 4-year resolution, respectively. ^10^Be was also measured at sub-annual resolution in the EGRIP (Eastern Greenland Ice core Project, 75°38′N, 36°00′W, 2704 m a.s.l.) ice core. Furthermore, lower resolution ^10^Be data from the GRIP (Greenland Ice core Project, 72°34′N, 37°37′W, 3029 m a.s.l., ~6 years resolution) and the EDML (EPICA Dronning Maud Land in Antarctica, 79°00′S, 0°04′E, 2892 m a.s.l., ~5 years resolution) ice cores are also presented. All records support the occurrence of an extreme SEP event around 9125 years BP (before present, i.e., before 1950 CE), i.e., 7176 BCE, that induced one of the largest short-term ^10^Be increase detected so far. We include ^14^C production data^24^ that, besides supporting our evidence of the occurrence of the SEP event, provide a chronological marker useful to synchronize the dating of the ice core and tree ring timescales. We further discuss the occurrence of the event within the 11-year solar cycle.

## Results and discussion

### ^10^Be and ^36^Cl data

**Fig. 1:**
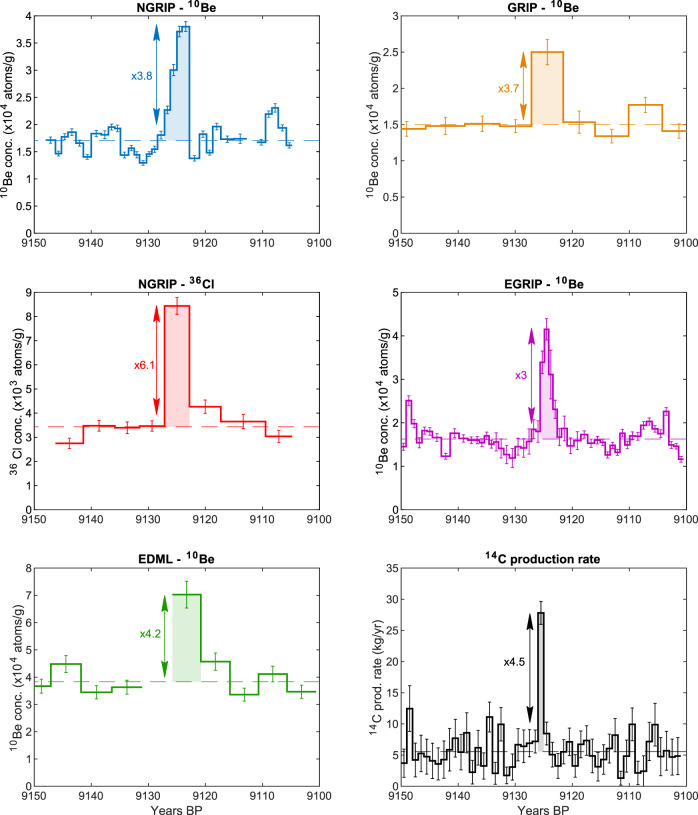
Cosmogenic radionuclide records for the 9125 years BP event. NGRIP and EDML data are shown on the left, GRIP and EGRIP ^10^Be data and ^14^C production rate^24^ are shown on the right. The baseline (average radionuclide concentration excluding the peak values) is shown as a dashed line. The error bar of each data point includes the measurement uncertainty. The enhancement factors (ratio between integrated enhancements in the radionuclide production and the baseline value) are noted next to the peak for each record.

^10^Be concentrations from the NGRIP, EDML, GRIP and EGRIP ice cores and ^36^Cl concentrations from the NGRIP ice core are displayed in Fig. 1. The data are plotted on the Greenland Ice core Time Scale (GICC05) with an adjustment of −54 years according to Adolphi and Muscheler^25^ (see Methods section). All the records show a sharp peak around 9125 years BP, in agreement with ^14^C production data^24^. The dashed lines in Fig. 1 represent the average concentration of ^10^Be and ^36^Cl over the displayed time windows (50 years for GRIP, EDML and EGRIP, ~40 years for NGRIP). This baseline concentration was calculated excluding the values of the peak (highest value for low-resolution GRIP and EDML records and values exceeding 2σ of the baseline around the highest value for high-resolution NGRIP and EGRIP records), thus considered to represent only the regular production rate changes, transport/depositional variability, and measurement scatter. The uncertainty of the baseline is calculated as the standard deviation of the data before and after the peak and also includes the measurement errors. The increase in radionuclide concentrations, which we consider to be solely caused by the production event, is determined for each record as the time-integrated ^10^Be and ^36^Cl concentration exceeding their respective baselines (integrated enhancement, represented by the colored area in Fig. 1). Here we consider only the relative enhancements. This approach is more robust as the absolute deposition (and increase) depends on a variety of factors that cannot be reliably quantified at the moment. These include, for example, spatial differences in the transport and deposition of ^10^Be^26^. The integrated enhancements were then used to calculate the enhancement factors (integrated enhancement divided by the baseline), i.e., relating the increased radionuclide deposition to the annual average radionuclide deposition before and after the event. In doing so, we obtain enhancement factors for ^10^Be of 3.85 ± 0.68 (NGRIP), 4.21 ± 1.10 (EDML), 3.74 ± 0.77 (GRIP) and 2.98 ± 0.70 (EGRIP). As for ^36^Cl, we find an enhancement factor of 6.09 ± 1.21. The baseline values, integrated enhancements and enhancement factors are listed in Table 1. Since the resolution of the ^36^Cl samples is 4 years, all four corresponding ^10^Be samples in the NGRIP record were considered in order to calculate the average ^10^Be value, integrated enhancement and enhancement factor for exactly the same period for ^10^Be and ^36^Cl. Averaging the ^10^Be enhancement factors of all four ice cores, we get an average ^10^Be enhancement factor of 3.69 ± 0.43. The imprints left in the ice cores by the event reveal a ^10^Be increase possibly larger than the one left by the 774/5 CE event (3.4 ± 0.3^16^, updated to 3 by Mekhaldi et al.^11^), but within the same uncertainty envelope. The shape of the peaks does not provide any additional robust information on the nature of the event, as they may also derive from a series of stochastic factors, such as climate and depositional effects^27^, stratosphere-troposphere exchange rates that are modulated seasonally^15,28,29^ and sampling issues. Figure 1 includes the ^14^C production data from Brehm et al.^24^, with an enhancement factor of 4.5 ± 0.5, about 20% higher than the average ^10^Be enhancement factor.

**Table 1:** Baseline value, integrated enhancement, and enhancement factor of each radionuclide record used.

Summary of results				
Ice core	Isotope	Baseline × 10^4^ atoms/g	Integrated enhancement × 10^4^ atoms/g	Enhancement factor
NGRIP	^10^Be	1.71 ± 0.25	6.58 ± 0.61	3.85 ± 0.68
	^36^Cl	0.36 ± 0.05	2.18 ± 0.28	6.09 ± 1.21
GRIP	^10^Be	1.50 ± 0.17	5.60 ± 0.96	3.74 ± 0.77
EDML	^10^Be	3.81 ± 0.52	16.07 ± 3.58	4.21 ± 1.10
EGRIP	^10^Be	1.63 ± 0.28	4.84 ± 0.84	2.98 ± 0.70

The baseline is calculated as the average concentration of each radionuclide excluding the peak values. The enhancement factors are calculated as the ratio between integrated enhancement over one year and baseline. Uncertainties are based on error propagation and include standard deviation of the baseline and measurement error.

### Spectral hardness

Theoretical calculations show that there is a tight link between the spectral hardness and the ^36^Cl/^10^Be ratio^30^ which has been used and supported in past ice core studies on solar storms^11,16,21^. The production rates of ^10^Be and ^36^Cl, relative to one another, are very sensitive to the energy spectrum of the SEPs reaching Earth, which leaves a specific signature in the radionuclide production enhancement ratio. That is, the production of ^10^Be by typical SEPs is maximal at ~200 MeV, whereas ^36^Cl production by SEPs peaks at ~30 MeV, due to a ^36^Cl production rate resonance effect for proton interaction with ^40^Ar^30^. This means that softer events, characterized by a higher proportion of lower energy particles, will trigger a relatively more enhanced production of ^36^Cl relative to ^10^Be compared to hard events, and are thus characterized by a larger ^36^Cl/^10^Be enhancement ratio. So far, GLE no.24 of August 1972, is the GLE with the softest spectrum to have ever been measured^9^, and theoretical estimates indicate that it caused an increase of only 1.2% in the global yearly ^10^Be production rate according to Mekhaldi et al.^11^ versus a 9.9% increase in ^36^Cl, leading to a ^36^Cl/^10^Be enhancement ratio of 8.6. On the other hand, GLE no.5 of February 1956 is the hardest GLE detected and caused an increase of about 5.1% in the global yearly ^10^Be production (vs. 8% in ^36^Cl), leading to a ^36^Cl/^10^Be excess ratio of 1.57^11^.

For the calculation of the ^36^Cl/^10^Be ratio, we used ^10^Be and ^36^Cl from NGRIP. This is justified by the fact that, at the same location, the non-production variability of ^10^Be and ^36^Cl records partly derives from the same factors such as snow accumulation influences on the radionuclide deposition. In addition, the NGRIP ^10^Be enhancement is very close to the average enhancement of the four ^10^Be records. Following the approach from O’Hare et al.^21^, a common ^36^Cl/^10^Be baseline was estimated. The ^36^Cl/^10^Be ratio was calculated for each ^36^Cl datapoint and the corresponding four ^10^Be samples (0.212 ± 0.038). The ^36^Cl baseline was then calculated from the ^10^Be baseline (^36^Cl baseline = 0.212 × ^10^Be baseline). As a consequence, the baseline variability and its uncertainty are considered only once as opposed to those of each radionuclide being estimated separately. As a result, we obtain a ^36^Cl/^10^Be excess ratio of 1.59 ± 0.38. This places the event in the category of a hard event (large fluxes of high energy protons) akin to GLE no.5 from February 1956.

### Fluence spectrum

The higher-resolution EGRIP data show that the ^10^Be peak lasts about 3 years, suggesting one or several events within short time occurred on a much shorter timescale. The production of ^10^Be nuclides by SEPs occurs higher in the stratosphere, relative to those produced by GCRs^11,30,31^, because the softer energy spectrum of the incident SEP particles hinders them from penetrating deep into the atmosphere. Upon production, ^10^Be binds to aerosols and has an average stratospheric residence time of 1–2 years^32^. As a result, the stratospheric ^10^Be signal can be assumed to be globally well mixed^33^. Modelling ^10^Be transport and deposition using a general circulation model, it has been shown that the well-mixed stratospheric ^10^Be is the dominant fraction of the radionuclides deposited in ice cores, representing 69% of the signal at GRIP^33^. Considering that we expect an enhanced SEP-induced production signal almost exclusively in the stratosphere, the overall effect is that we can consider the Greenland ^10^Be records to be close to the global average production in terms of relative changes for SEP events. We assume that the same holds true for ^36^Cl that has a similar stratospheric residence time as ^10^Be^34^. ^10^Be peaks may sometimes be linked to strong stratospheric eruptions^14,35^, but these would not be associated with peaks in ^14^C and ^36^Cl records, allowing us to rule out this hypothesis as a cause for the considered event.

As mentioned above, there is a ~20% difference between the relative ^10^Be and ^14^C enhancements. A similar disagreement was found for 774/5 CE and 993/4 CE events^16^. It is difficult to pinpoint the exact reason for this potentially systematic bias. It could be explained by the large uncertainties that characterize the radionuclide production yield functions^11^. In particular, the different ^14^C yield functions are characterized by a large spread for E < 500 MeV^36^. It could possibly also be explained by the different geochemical behavior of ^14^C and ^10^Be. However, we find no evidence of a polar bias^37^, as a larger ^10^Be amplitude would be expected due to the enhanced polar production rate during solar storms. This is opposite to our observations. The slightly smaller amplitude in the ^10^Be increase factor from the EGRIP record compared to the other records could also be due to some smoothing effect due to the sampling method for the CFA (Continuous Flow Analysis) samples (see Methods section).

Assuming that the radionuclide records from the Greenland ice cores are varying proportionally to the global production rates of ^10^Be and ^36^Cl we can estimate the fluence and magnitude of the 9125 years BP event by assessing the relative increase of ^36^Cl in NGRIP and ^10^Be in the various ice cores used here. To estimate the integrated fluence of the event, we compared the observed ice core ^36^Cl/^10^Be ratio to ratios obtained from modeling the global production rate of radionuclides caused by known modern events and selected those that agreed within uncertainty as the modern analogs. Mekhaldi et al. (2021)^11^ reassessed the global ^10^Be and ^36^Cl production rates by GCRs and associated increases caused by GLEs throughout 1951–2016. The contribution of the GLEs to the mean global production rate of ^10^Be and ^36^Cl was calculated by taking into account the spectral parameters from Raukunen et al.^8^ and the production functions from Poluianov et al.^31^. The selected events are listed in Table 2 with their corresponding ^36^Cl/^10^Be ratios and fluences.

**Table 2:** Information about the modern GLEs with spectral shapes agreeing with the ^36^Cl/^10^Be ratio for the 9125 years BP event: the ^10^Be production increase factor of each reference GLE relative to the GCR baseline for Φ = 650 MV, fluences (protons/cm^2^) above 30, 200 and 430 MeV^8,9^.

Summary of GLEs and SEP events discussed in this study and associated radionuclide production						
Event	GLE no.	^10^Be production increase factor (X)	^36^Cl/^10^Be enhancement ratio	F_30_ (protons/cm^2^)	F_200_ (protons/cm^2^)	F_430_ (protons/cm^2^)
23-Feb-56	5	5.10E − 02	1.57	1.42E + 09	1.21E + 08	3.03E + 07
04-May-60	8	2.40E − 04	1.45	4.84E + 06	5.31E + 05	1.50E + 05
28-Jan-67	16	1.50E − 03	1.97	8.52E + 07	4.36E + 06	7.41E + 05
30-Mar-69	21	6.00E − 04	1.96	3.25E + 07	1.66E + 06	2.54E + 05
24-Sep-77	29	4.70E − 04	1.94	2.53E + 07	1.38E + 06	2.30E + 05
15-Nov-89	46	1.00E − 04	1.97	5.20E + 06	3.15E + 05	4.18E + 04
26-May-90	49	4.80E − 04	1.87	2.03E + 07	1.60E + 06	2.03E + 05
20-Jan-05	69	6.80E − 03	1.93	3.29E + 08	2.21E + 07	2.89E + 06
9125 years BP	-	3.69 ± 0.43	1.59 ± 0.38	1.64 (±0.53)E + 11	1.06 (±0.19)E + 10	1.80 (±0.35)E + 09
774/5 CE	-	3.4 ± 0.3	1.8 ± 0.2	8.3 (±4.5)E + 10	N/A	N/A
993/4 CE	-	1.2 ± 0.2	2.1 ± 0.4	3.3 (±1.8)E + 10	N/A	N/A
660 BCE	-	2.52 ± 0.91	1.4 ± 0.3	6.9 (±3.8)E + 10	N/A	N/A

The fluences of the 9125 years BP event are calculated as the average of the fluences of the scaled-up spectra. The uncertainties of the fluence estimates include the uncertainties of the ^10^Be enhancement factors and the standard deviation of the scaled fluence spectra. The ^36^Cl/^10^Be ratio and fluence above 30 MeV for 774/5 CE, 993/4 CE, and 660 BCE^16,21^ are also reported for comparison. The fluences of these events have been updated by Mekhaldi et al.^11^.

**Fig. 2:**
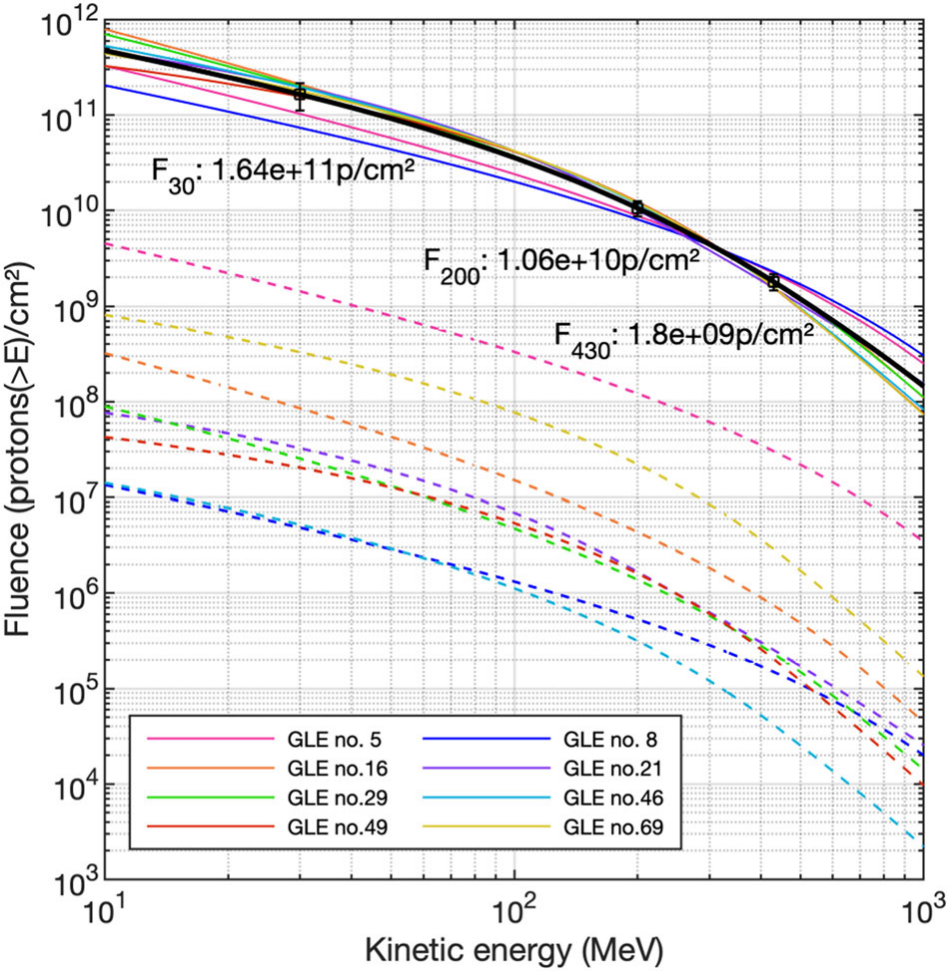
Event-integrated fluence spectra (assuming an average Φ = 650 MV). The dashed curves represent the original spectra of the modern events that fit the ^36^Cl/^10^Be ratio of the 9125 years BP event. The scaled spectra are shown as continuous lines. The black line shows the average fluence spectrum. The fluences above 30, 200, and 430 MeV (F_30_, F_200_ and F_430_) of the average spectrum are reported. The uncertainties of the fluence estimates include the uncertainty of the ^10^Be enhancement factor and the standard deviation of the scaled spectra.

To assess the fluence of the event, we consider the average ^10^Be enhancement factor for the 9125 years BP event (3.69 ± 0.43) and compare it to the modeled increase in ^10^Be for the selected modern events (average enhancement factor of the event divided by the enhancement factor of the known GLEs). The obtained coefficients were used to scale the fluence spectra of the modern events. For example, we find that GLE no.5 (1956) caused an annual ^10^Be production increase (X_56_) of 5.1%^11^. We thus multiplied the fluence spectrum of GLE no.5 by the coefficient of 3.69/X_56_, i.e., a factor of 72 ± 8. The uncertainties of the scaling coefficients include the uncertainties related to the ^10^Be enhancement factors of both modern events and the 9125 years BP SEP event. The fluences (F_30_, F_200_, F_430_) of the modern events used were taken from Cliver et al.^9^, based on the spectral parameters provided by Raukunen et al.^8^. The original spectra of the events that fit the ^36^Cl/^10^Be ratio are shown as dashed curves in Fig. 2 whereas the continuous curves show the spectra of the events multiplied by the corresponding scaling coefficient. Finally, we also report the average spectrum of the newly-calculated spectra in the Figure (black curve), with a F_30_ of 1.64 (±0.53) × 10^11^ protons/cm^2^, thus possibly up to two orders of magnitude larger than GLE no. 5, the strongest ground level enhancement to date. The uncertainty of the fluence (>30, 200, and 430 MeV) estimates reported in Table 2 include the uncertainties related to the ^10^Be enhancement factors and the standard deviation of the scaled fluence spectra. It is possible that the ancient event was characterized by different spectral shape than the smaller modern events, as the ^36^Cl/^10^Be ratio provides information on the spectral hardness (relationship between ~30 MeV and ~200 MeV) but no additional details on the spectrum. The fluence (>E) estimates provided in Table 2 should thus be considered with caution, though they illustrate well the extreme nature of the 9125 years BP event. If the ^14^C enhancement factor is used in the calculations, we find F_30_ is about 20% higher than using ^10^Be data from ice cores. This shows that our ^10^Be-derived fluence reconstruction provides a rather conservative estimate of the protons flux generated during the SEP event. Applying the same methodology but using the updated spectra from Koldobskiy et al.^38^, we find a F_30_ of 1.27 (±0.48) × 10^11^, thus agreeing within uncertainties. The fluence spectra of modern GLEs from Koldobskiy et al.^38^ have different spectral shapes than the ones from Raukunen et al.^8^, with lower fluences around 30 MeV, leading to this difference. At higher energies, where ^10^Be production is the most efficient (200–300 MeV), the spectra are very similar, leading to almost identical F_200_. A comparison of the fluence spectra of the 9125 years BP event modeled using the fluence spectra of modern GLEs from Koldobskiy et al.^38^ and Raukunen et al.^8^ is shown in supplementary Fig. 1.

For the calculation of the increase in the global production rate of cosmogenic radionuclides caused by GLEs since the 1950s and with respect to production by GCRs, Mekhaldi et al.^11^ considered a baseline Φ = 650 MV, representing the mean solar modulation during the Space Age^39^. However, the solar modulation during the studied time period was likely lower^40,41^. This is, however, uncertain as the different radionuclide-based reconstructions differ significantly^40,42^. By averaging the solar modulation values presented by Vonmoos et al.^40^ and Steinhilber et al.^41^ over 9100–9150 years BP (values of 260 MV and 337 MV respectively), we repeated the calculations with a baseline Φ = 300 MV. If we take, for example, GLE no.5, we then find a scaling coefficient (3.69/X_56_) of 95 (±10) (vs. 72(±8) at Φ = 650 MV). This would thus imply a F_30_ of 2.17 (±0.81) × 10^11^ protons/cm^2^ making this event larger than the 774/5 CE event^11,16,17^. On the other hand, if we consider the solar modulation values presented by Roth and Joos^42^, the average value over the targeted period is closer to the Space Age value (at Φ = 615 MV). Using this value the scaling coefficients are 2% higher than at Φ = 650 MV, which leads only to a minor change for the fluence. A correction for a possible variation in the geomagnetic shielding is not required, as both GCRs and SEPs are affected similarly, leading to no significant changes in the relative production rate enhancement of cosmogenic radionuclides for SEP events^11^. Our fluence estimates in Table 2 are therefore rather lower limits considering this uncertainty in the calculation.

### Timing within the 11-year solar cycle

**Fig. 3:**
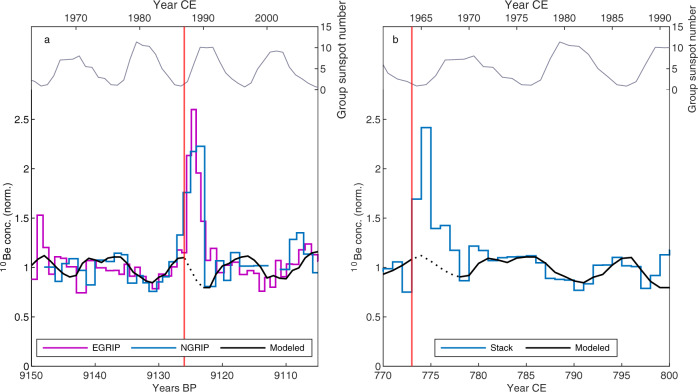
Relationship between the solar 11-year cycle and the occurrence of the solar energetic particle events of 9125 years BP and 774/5 CE. **a** The normalized ^10^Be records from NGRIP and EGRIP from 9150 to 9105 years BP (see legend) compared to the normalized ^10^Be annual production rate modeled from neutron monitor data^11^ for the period 1963–2008 (black line), showing the best correlation coefficients (for details see main text, EGRIP: *r* = 0.45, *p* < 0.01; NGRIP: *r* = 0.51, *p* < 0.01). The top panel shows the group sunspot number corresponding to the period of the neutron monitor-based production rate^49^. **b** The same comparison for the average ^10^Be data from ice cores from Greenland (NEEM S1, NGRIP, and Tunu) and Antarctica (WAIS) from 770 to 800 CE (blue line) to the period 1961–1991 (neutron-monitor based production rate, black line), showing the best correlation coefficient (for details see main text, *r* = 0.69, *p* < 0.01). NEEM, NGRIP, and Tunu from Greenland and WAIS from Antarctica were normalized to their baseline (average ^10^Be concentration excluding the peak). The red line indicates the estimated onset of the event. The timescales are independently matched^25,59^, i.e., not matched to get a fit for the ^10^Be peak.

The relationship between the occurrence of SEP events and solar activity has been discussed for the Space Age period (1950s-Present). It has been observed that the majority of these events occur during an active phase, around solar maxima^43–46^. Performing the same analysis on the paleoevents has so far been hindered by the coarser resolution of the radionuclide records and the scarcity of events discovered. Nevertheless, Sukhodolov et al.^47^ tried to model the 11-year solar cycle around the 774/5 CE event, showing that the SEP event likely occurred near a solar minimum, when ^10^Be concentration peaks, in agreement with the data shown by Mekhaldi et al.^16^ and Sigl et al.^48^. In this study, we compare the modeled ^10^Be annual production rate caused by GCRs and modulated by the 11-year solar cycle from Mekhaldi et al.^11^ to our high-resolution ^10^Be data from EGRIP and NGRIP around 9125 years BP and to ^10^Be data from 774/5 CE^16^ to investigate the occurrence of the two events within the solar 11-year cycle. In this analysis we investigate the best fit between the normalized ^10^Be data from ice cores around the 9125 years BP and 774/5 CE events (excluding the peaks) and the normalized modeled production rate of ^10^Be from the last 70 years inferred from neutron monitor data (Fig. 3). The fitting has been carried out by shifting the globally averaged and normalized ^10^Be production rate modeled from neutron monitors in time versus our normalized data from NGRIP and EGRIP around 9125 years BP and the stack of ^10^Be records for 774/5 CE^16^ (excluding the peak). The selected scenarios are those for which we obtained statistically significant results (*p* < 0.05, computed using a t-test). We note that the 11-year solar cycle appears to be well-preserved in the records from NGRIP and EGRIP around 9125 years BP (see Fig. 3a). In general, the ice core data indicate a good agreement with theoretically expected production variations of ^10^Be by GCRs, with a slight mismatch between 9140 and 9145 years BP. We find that four complete solar cycles with a duration of 10-11 years are observable (approximately 9105–9116, 9116–9126, 9126–9137, 9137–9148 years BP, from peak to peak in the ^10^Be concentration). From Fig. 3 we can also point out that the relative amplitudes (of about ±20%) of the variations of the 11-year solar cycles match the amplitude of the expected ^10^Be global production rates for modern solar modulation (here 1963–2008, Fig. 3a). With this fitting we obtain the best correlation coefficients (EGRIP: *r* = 0.45, *p* < 0.01; NGRIP: *r* = 0.51, *p* < 0.01, stack: *r* = 0.52, *p* < 0.01, where r is Pearson correlation coefficient). The suggested timing of the SEP event is indicated with a red line in Fig. 3, as inferred from the ^14^C production data^24^, between the growing seasons of 9126 and 9125 years BP. The yearly group sunspot number from Svalgaard et al. (2016)^49^ corresponding to the neutron monitor-inferred ^10^Be production rate is also shown in Fig. 3. The SEP event signal and the timing of the event could be affected by a delay of one year due to the residence time of stratospheric ^10^Be^32^. The data indicate that the event likely occurred close to the solar minimum. Similar results are obtained for the 774/5 CE event. Figure 3 (panel b) shows the normalized ^10^Be record from four ice cores from Greenland and Antarctica around the aforementioned event. The records from the Greenland ice cores NGRIP, NEEM and Tunu as well as the Antarctic ice core WAIS were normalized to their baseline (average concentration excluding the peak) and averaged to obtain a global stack. In this way, the noise inherent to the data can be significantly reduced owing to the availability of several radionuclide records, allowing the 11-year solar cycle to be clearly identifiable. Similarly to the period around the 9125 years BP event, the relative amplitudes of the wiggles in ^10^Be concentrations related to the 11-year solar cycles match the amplitude of the expected ^10^Be global production rates from 1961 to 1991 (*r* = 0.69, *p* < 0.01). In agreement with the results from Sukhodolov et al.^47^, we find that the 774/5 CE event occurred during a period of low solar activity. The same analysis was carried out on ^10^Be data around the 993/4 CE^16^ and 660 BCE^21^ events, but no significant results were obtained due to the noise inherent to the data (see supplementary Fig. 2).

### Timescale implications

The identification of a synchronous peak in ^10^Be from ice cores and in ^14^C from tree rings provides a valuable global time-marker. First, the present work confirms the validity of the transfer function proposed by Adolphi and Muscheler^25^, i.e., the synchronization of the GICC05 and IntCal time-scales based on common centennial-scale variations in the production rates of ^10^Be and ^14^C in ice cores and tree rings leading to an adjustment of −54 years (±6 years) to the GICC05 time scale. Secondly, the independent discovery of a peak in radiocarbon and ^10^Be allows the reduction of the time-scale synchronization uncertainty around that time to about one year connected to the sampling uncertainty and the ^10^Be residence time. Therefore, our results support the 54 years offset but reduce the uncertainty for the match between the Greenland ice core time scale to the absolute dendrochronologically determined IntCal ^14^C time scale from an estimated 6 years^25^ to only one year around 9125 years BP.

To conclude, the data presented here provide evidence for an (or a series of) extreme SEP event(s) around 9125 years BP, showing one of the largest relative ^10^Be enhancement detected so far in ice cores. Furthermore, the reconstruction of the ^36^Cl/^10^Be enhancement ratio suggests that this SEP event was characterized by a very hard spectrum and that it was similar or even larger than the 774/5 CE event in terms of fluence (>30 MeV). This thus further pushes the magnitude of a potential worst-case scenario for SEP events. We also provide evidence that the 9125 years BP and 774/5 CE events occurred near a solar minimum, contrary to expectations but in agreement with previous studies conducted on the 774/5 CE event^47^. Additional events need to be discovered and studied at similarly high resolution in order to robustly assess whether there is a consistent pattern in the occurrence of extreme SEP events in relation to the 11-year solar cycle and solar activity levels in general, and the probability of occurrence of such extreme events. Identifying whether there exists a relationship between solar activity and occurrence of extreme solar storm events is fundamental for the planning of space missions, in order to minimize the risk for space technology and for the health of astronauts. We also provide a valuable time marker to more precisely constrain the dating of the ice cores and allowing the reduction of the timescale uncertainty.

## Methods

### Ice core data

NGRIP ice was sampled at an equidistant resolution of 11 cm (5 samples per 55 cm ice core bag), corresponding to an average temporal resolution of ~1 year for ^10^Be samples. Due to the lower ^36^Cl concentrations, 4 samples were combined for the measurement of ^36^Cl, resulting in average temporal resolution of ~4 years.

The EGRIP Continuous Flow Analysis (CFA) samples were collected at Climate and Environmental Physics (University of Bern) by sampling the outer part of the CFA ice samples, otherwise discarded^50^. The EGRIP ^10^Be samples have been collected at an average temporal resolution of 0.85 years. The excess water was collected in 50 ml vials containing ^9^Be and ^35/37^Cl carriers. The water flows through a small plastic tube connected to the CFA system. This procedure implies an uncertainty of a few centimeters in relating the ^10^Be sample to the depth of the continuously melting ice stick. Smoothing of the CFA chemistry data is typically on the order of months^51,52^ due to the mixing in the analysis channels. The ^10^Be line very likely has lower smoothing due to the very high flowrate of the segmented air/water mix in the line.

The GRIP ^10^Be record is available for this period at a temporal resolution of ~6 years^53,54^, while EDML ^10^Be data are available at a resolution of ~5 years.

^10^Be concentrations from EGRIP CFA samples were blank corrected (average blank ^10^Be/^9^Be ratio: ~10% of the sample ^10^Be/^9^Be ratio, average ^10^Be/^9^Be ratio of the blank samples: 0.013 **×** 10^−12^). The other records were not corrected as the blank corrections were considered to be negligible (average blank ^10^Be/^9^Be ratio for NGRIP: 2% of the sample ^10^Be/^9^Be ratio, average ^10^Be/^9^Be ratio of the blank samples: 0.008 **×** 10^−12^; average ^36^Cl/Cl ratio for NGRIP: 3% of the sample ^36^Cl/Cl ratio, average ^36^Cl/Cl ratio of the blank samples: 0.001 **×** 10^−12^), in agreement with previous studies^53^.

### ^10^Be and ^36^Cl extraction from ice samples

NGRIP ice samples (~175 g) were prepared using ion exchange chromatography following the procedure described by Adolphi et al.^55^ with the addition of 0.150 mg of ^9^Be carrier. The smaller EGRIP excess water samples (~50 g) were prepared without the use of ion exchange chromatography with the addition of 0.100 mg of ^9^Be carrier. Be(OH)_2_ was directly precipitated with NH_4_OH. After centrifugation, the precipitate was transferred to a quartz crucible and the same procedure as Adolphi et al.^55^ was then followed for the oxidation to BeO and pressing for AMS measurement.

^36^Cl preparation was carried out following the procedures from Delmas et al.^56^ with the addition of 4 mg Cl carrier. The measurement of ^10^Be and ^36^Cl was carried out at the Laboratory of Ion Beam Physics at ETH, Zurich (Switzerland) using Accelerator Mass Spectrometry^57^. The measured ^10^Be/^9^Be ratios were normalized to the ETH Zurich in house standards S2007N and S2010N^57^, which were both calibrated relative to the ICN 01-5-1 standard (^10^Be/^9^Be = 2.709 × 10^−11^ nominal)^58^. The measured ^36^Cl/Cl ratios were normalized to the ETH Zurich in house standard K382/4N^57^ with a nominal value of (17.36 ± 0.34) × 10^−12^.

### Timescale

GICC05 timescale was corrected according to Adolphi & Muscheler^25^ with an adjustment of −54 years (±6 years). Since the EDML timescale has been matched to GICC05^59^, the same timescale adjustment has been applied to the EDML ice core to match the IntCal chronology^25^.

## Supplementary information

Supplementary information

## Data Availability

The ^10^Be and ^36^Cl data generated in this study are provided in the Supplementary Information. Source data are provided with this paper.

## Source data

Source Data
