# Supplementary material for: Cosmogenic radionuclides reveal an extreme solar particle storm near a solar minimum 9125 years BP
Source: Nat Commun. 2022 Jan 11;13:214. doi: 10.1038/s41467-021-27891-4 (PMC8752676; doi:10.1038/s41467-021-27891-4)
Supplement: Supplementary file 1 — Supplementary information [file 41467_2021_27891_MOESM1_ESM.pdf]

Supplementary Information for 'Cosmogenic radionuclides reveal an extreme solar particle storm near a solar minimum 9125 years BP'

Chiara I. Paleari[1*], Florian Mekhaldi[1,2], Florian Adolphi[3], Marcus Christl[4], Christof Vockenhuber[4], Philip Gautschi[4], Jürg Beer[5], Nicolas Brehm[4], Tobias Erhardt[3,6], Hans-Arno Synal[4], Lukas Wacker[4], Frank Wilhelms[3,7] and Raimund Muscheler[1]

[1]Department of Geology – Quaternary Sciences, Lund University, 22362, Lund, Sweden

[2]British Antarctic Survey, Ice Dynamics and Paleoclimate, Cambridge, CB3 0ET, UK

[3] Alfred-Wegener-Institut Helmholtz-Zentrum für Polar- und Meeresforschung, 27570 Bremerhaven, Germany

[4]Laboratory of Ion Beam Physics, ETH Zürich, 8093 Zürich, Switzerland

[5]Department of Surface Waters, Swiss Federal Institute of Aquatic Science and Technology, 8600 Dübendorf, Switzerland

[6]Climate and Environmental Physics, Physics Institute and Oeschger Centre for Climate Change Research, University of Bern, 3012 Bern, Switzerland

[7]Department of Crystallography, Geoscience Centre, University of Göttingen, Göttingen, Germany

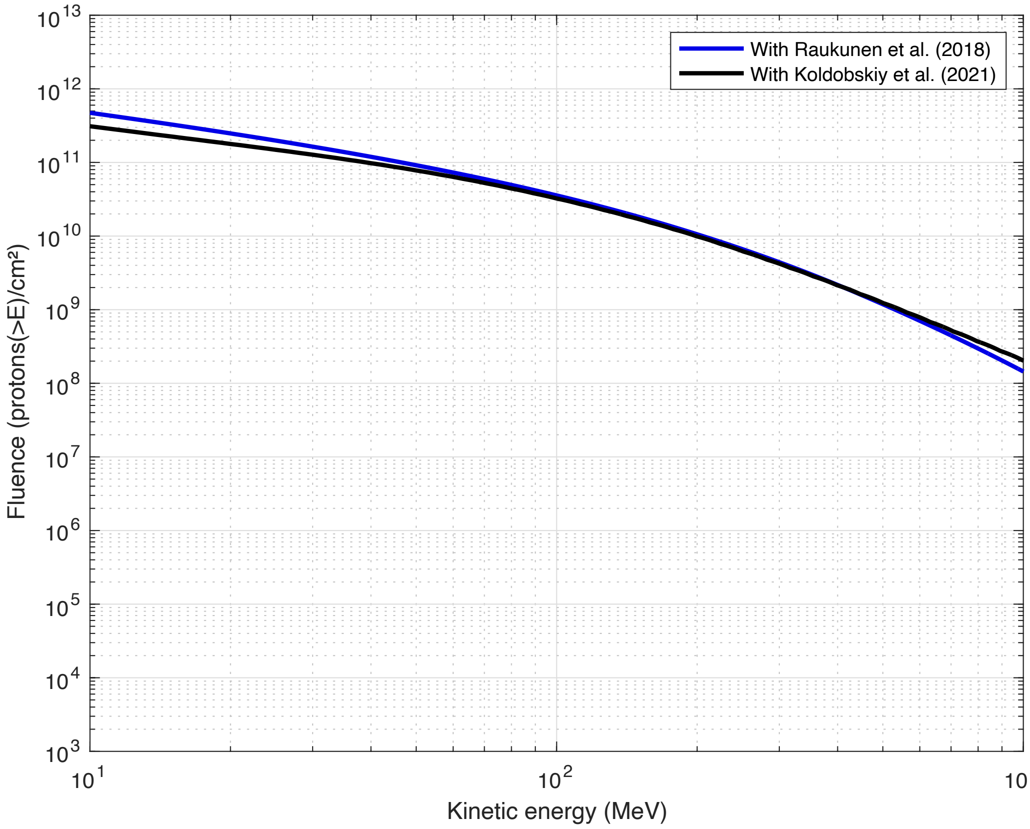

Supplementary Figure 1. Fluence spectra reconstructions for the 9125 years BP event using the spectra of modern GLEs from Koldobskiy et al. (2021)[1], in black, and from Raukunen et al. (2018)[2], in blue.

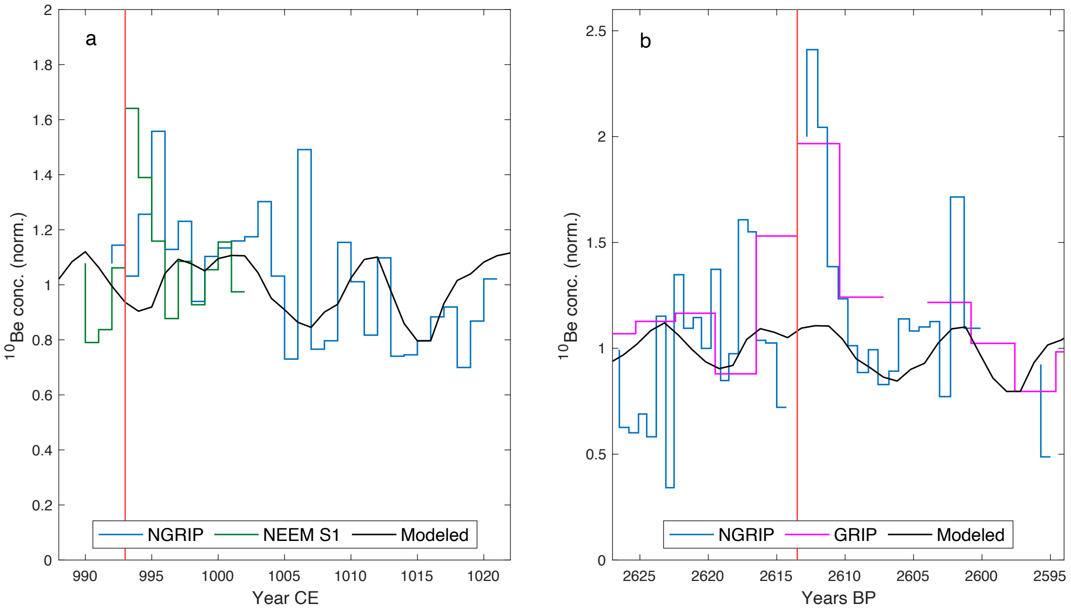

Supplementary Figure 2. Attempt at identifying the relationship between the solar 11-year cycle and the occurrence of the solar energetic particle events of 993/4 CE[3] and 2610 years BP[4]. Panel (a) shows the normalized ^{10}Be records from NGRIP (blue) and NEEM S1 (green) around the 993/4 CE event compared to the normalized ^{10}Be annual production rate modeled from neutron monitor data[5] (period 1966-1994 CE). Panel (b) shows the same comparison to the average ^{10}Be data from ice cores from NGRIP (blue) and GRIP (magenta) for the 2610 years BP event with the normalized ^{10}Be annual production rate modeled from neutron monitor data[5] (period 1961-1994). All records are normalized to their baseline (average ^{10}Be concentration excluding the peak). The red line indicates the estimated onset of the event.

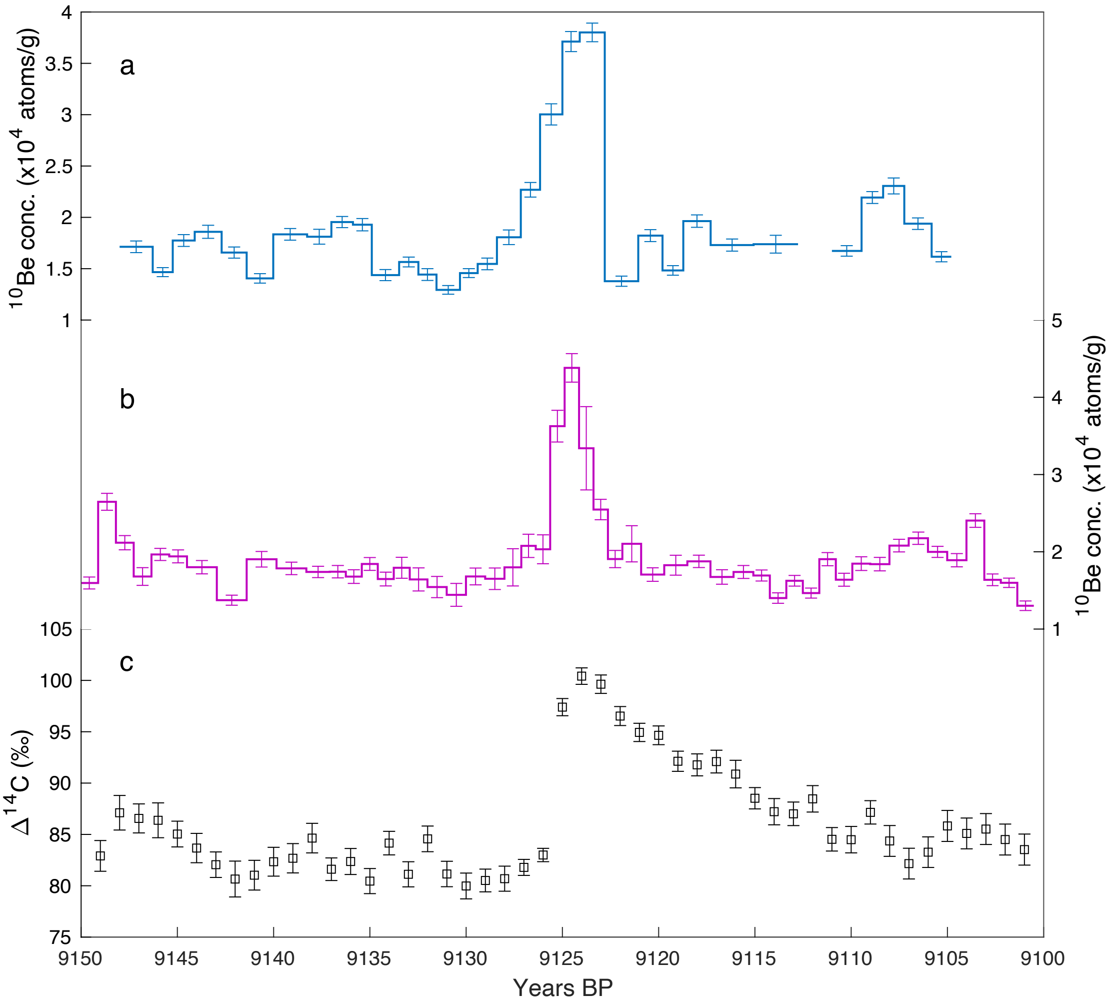

Supplementary Figure 3. Comparison between ^{10}Be and radiocarbon records. The NGRIP ^{10}Be record is shown in panel a), the EGRIP ^{10}Be record in panel b). The error bar of each data point includes the measurement uncertainty. Panel c) shows the mean Δ^{14}C record from Brehm et al. (2021)[6]. The records show synchronous ^{10}Be and ^{14}C peaks after an adjustment of the GICC05 timescale by -54 years (±6 years)[7].

References

1. Koldobskiy, S., Raukunen, O., Vainio, R., Kovaltsov, G. A. & Usoskin, I. New reconstruction of event-integrated spectra (spectral fluences) for major solar energetic particle events. *Astron. Astrophys.* **647**, 1–16 (2021).
2. Raukunen, O. *et al.* Two solar proton fluence models based on ground level enhancement observations. *J. Sp. Weather Sp. Clim.* **8**, 1–19 (2018).
3. Mekhaldi, F. *et al.* Multiradionuclide evidence for the solar origin of the cosmic-ray events of 774/5 and 993/4. *Nat. Commun.* **6**, 1–8 (2015).
4. O'Hare, P. *et al.* Multiradionuclide evidence for an extreme solar proton event around 2,610 B.P. (~660 BC). *Proc. Natl. Acad. Sci.* **116**, 201815725 (2019).
5. Mekhaldi, F., Adolphi, F., Herbst, K. & Muscheler, R. The Signal of Solar Storms Embedded in Cosmogenic Radionuclides: Detectability and Uncertainties. *J. Geophys. Res. Sp. Phys.* **126**, (2021).
6. Brehm, N. *et al.* Tree-rings reveal two strong solar proton events in 7176 and 5259. *Nat. Commun.* (2021). Preprint at doi:10.21203/rs.3.rs-753272/v1
7. Adolphi, F. & Muscheler, R. Synchronizing the Greenland ice core and radiocarbon timescales over the Holocene-Bayesian wiggle-matching of cosmogenic radionuclide records. *Clim. Past* **12**, 15–30 (2016).
